# Unveiling the spatial distribution and transboundary pathways of FMD serotype O in Western China and its bordering countries

**DOI:** 10.1371/journal.pone.0306746

**Published:** 2024-08-16

**Authors:** Shuang Zhang, Rong Chai, Yezhi Hu, Fekede Regassa Joka, Xiaodong Wu, Haoning Wang, Xiaolong Wang

**Affiliations:** 1 Center of Conservation Medicine & Ecological Safety, Northeast Forestry University, Harbin, Heilongjiang Province, P. R. China; 2 The Key Laboratory of Wildlife Diseases and Biosecurity Management, Harbin, Heilongjiang Province, P. R. China; 3 Ethiopian Wildlife Conservation Authority, Addis Ababa, Ethiopian; 4 China Animal Health and Epidemiology Center, Qingdao, Shandong Province, P. R. China; 5 School of Geography and Tourism, Harbin University, Harbin, Heilongjiang Province, P. R. China; 6 Heilongjiang Cold Region Wetland Ecology and Environment Research Key Laboratory, Harbin University, Harbin, Heilongjiang Province, P. R. China; Sudan University of Science and Technology, College of Veterinary Medicine, SUDAN

## Abstract

Foot-and-mouth disease (FMD) is a severe, highly contagious viral disease of livestock that has a significant economic impact on domestic animals and threatens wildlife survival in China and border countries. However, effective surveillance and prevention of this disease is often incomplete and unattainable due to the cost, the great diversity of wildlife hosts, the changing range and dynamics, and the diversity of FMDV. In this study, we used predictive models to reveal the spread and risk of FMD in anticipation of identifying key nodes to control its spread. For the first time, the spatial distribution of FMD serotype O was predicted in western China and border countries using a niche model, which is a combination of eco-geographic, human, topographic, and vegetation variables. The transboundary least-cost pathways (LCPs) model for ungulates in the study area were also calculated. Our study indicates that FMD serotype O survival is seasonal at low altitudes (March and June) and more sensitive to temperature differences at high altitudes. FMD serotype O risk was higher in Central Asian countries and both were highly correlated with the population variables. Ten LCPs were obtained representing Pakistan, Kazakhstan, Kyrgyzstan, and China.

## Introduction

Foot-and-mouth disease is considered to be one of the highly contagious viral diseases that is caused by foot-and-mouth disease virus (FMDV) [1]. FMDV is a single stranded positive sense RNA virus, belonging to the genus *Aphthovirus* within the family *Picornaviridae*. FMDV has seven serotypes which are unevenly distributed around the world, namely A, O, C, Asia1, SAT1, SAT2, and SAT3 [2]. FMD affects cloven hoofed domestic animals such as cattle, sheep, goats, and pigs [2] and more than 70 wild animals such as impala (*Aepyceros melampus*) [3] and mountain gazelles (*Gazella gazelle*) [4]. Transmission occurs through contact with infected animals, their secretions and excreta, animal products, aerosolized droplets, and mechanical vectors [5, 6]. The manifestations of FMD and susceptibility to the disease vary according to the animal species and virulence of the virus strain [6, 7]. Airborne transmission, difficult for humans to control, is one of the mechanisms for spreading FMDV [8]. FMDV airborne transmission is much less compared to direct contact (same drinking point), and the conditions that constitute its transmission are more complex [9]. With a large dose of virus, the area of transmission needs to maintain a stable wind direction and speed, no precipitation, and a certain number of livestock nearby [8, 10]. The main clinical features of FMD infection are blisters and ulcers on the mucous membranes of the mouth, nose, feet, and teats, accompanied by decreased milk production and loss of appetite, as well as extensive necrosis of tissues in the pups, resulting in high mortality [2, 6, 7].

This disease has been reported in most parts of the world, with the exception of Greenland, Iceland, New Zealand, and smaller islands in Oceania. Outbreaks have primarily occurred in Asia, Africa and parts of Europe adjacent to Asia [11]. FMD serotypes O, A, and Asia 1 were first reported in 1958 in China. Subsequently, serotype O (O/Akesu/CHA/58) and A (A/XJ/KT/CHA/58) isolates were collected from Xinjiang Uygur Autonomous Region, while serotype Asia 1 (Asia 1/YN/BS/CHA/58) isolates were obtained from Yunnan Province [12]. There have been no reports of Asia 1 since 2009. FMD serotypes O and A are currently the most prevalent. The frequency of outbreaks serotype O are significantly more common than FMD serotype A [13].

In addition, wild animals play an important and complex role in the widespread transmission of this disease. Wild animals serve as indicators of pathogen circulation during FMD transmission and may also be bridge hosts or even maintenance hosts for some infections [14]. The African buffalo *(Syncerus caffer*) is the best-known reservoir host of FMD in the wild, particularly in Sub-Saharan Africa [15]. Other wild ruminants, like the impala (*Aepyceros melampus*), may also play a role in locally maintaining FMDV, especially at certain population densities [16]. Wildlife disease control options include prevention measures (biosecurity and movement control, including fencing), population control (targeted or random), and vaccination [14, 17]. However, the implementation of these prevention is generally incomplete and unattainable factors such as cost, the vast diversity of wildlife hosts, changing ranges and dynamics, and the continued expansion and the increasing diversity of animal diseases [14]. Additionally, the risk of FMD transmission between wild ruminants and livestock may increase as they share more habitats and water sources [18].

Given these issues, the impact of FMD on the global economy, especially in China, is staggering. The frequency of FMD outbreaks and the number of affected animals and species will have a lasting impact on FMD in endemic countries [19]. The visible annual impact of FMD in terms of production losses and vaccination in endemic areas globally amounts to US$6.5 to US$21 billion [20]. In China alone, this impact ranges from US$2.5 billion to US$7 billion [20]. In response to the recurrent development of FMD outbreaks to establish national and regional action plans and support, the FAO has developed a long-term regional approach for the progressive control of FMD, called the West Eurasia FMD Control—Roadmap [21]. However, the current challenges in controlling FMD are enormous, including the virus’s ability to infect many domesticated and wild species, due to its extreme infectivity [21]. This necessitates deeper insight into the virus’s spatial prevalence characteristics.

Based on the above-mentioned challenges, our study aims to gain a deeper understanding and predict the transmission pattern of FMD. FMD serotype O has been reported in all Central Asian countries, with the greatest risk of cross-border transmission occurring along the border between China and Russia [22]. Based on the abundance of domesticated and wild animal hosts in the region, coupled with its complex and harsh geography, surveillance and prevention of FMD are extremely challenging. Therefore, it is crucial to focus on risk prediction and monitoring of potential FMD transmission in western China and Central Asian countries.

We hypothesized that interspecies transmission of FMD serotype O occurs in cloven-hoofed animals in western China and across its borders, which forms the basis for the transboundary transmission of FMD. We used a maximum entropy model (MaxEnt) to predict the risk of FMD serotype O in western China and Central Asian countries. Additionally, we utilized the Least—Cost Path model to predict the migration pathways of ungulates between areas contaminated with FMD serotype O. This approach reveals potential FMD serotype O cross-border pathways between western China and neighboring countries.

## Material and methods

### Study area

**Fig 1:**
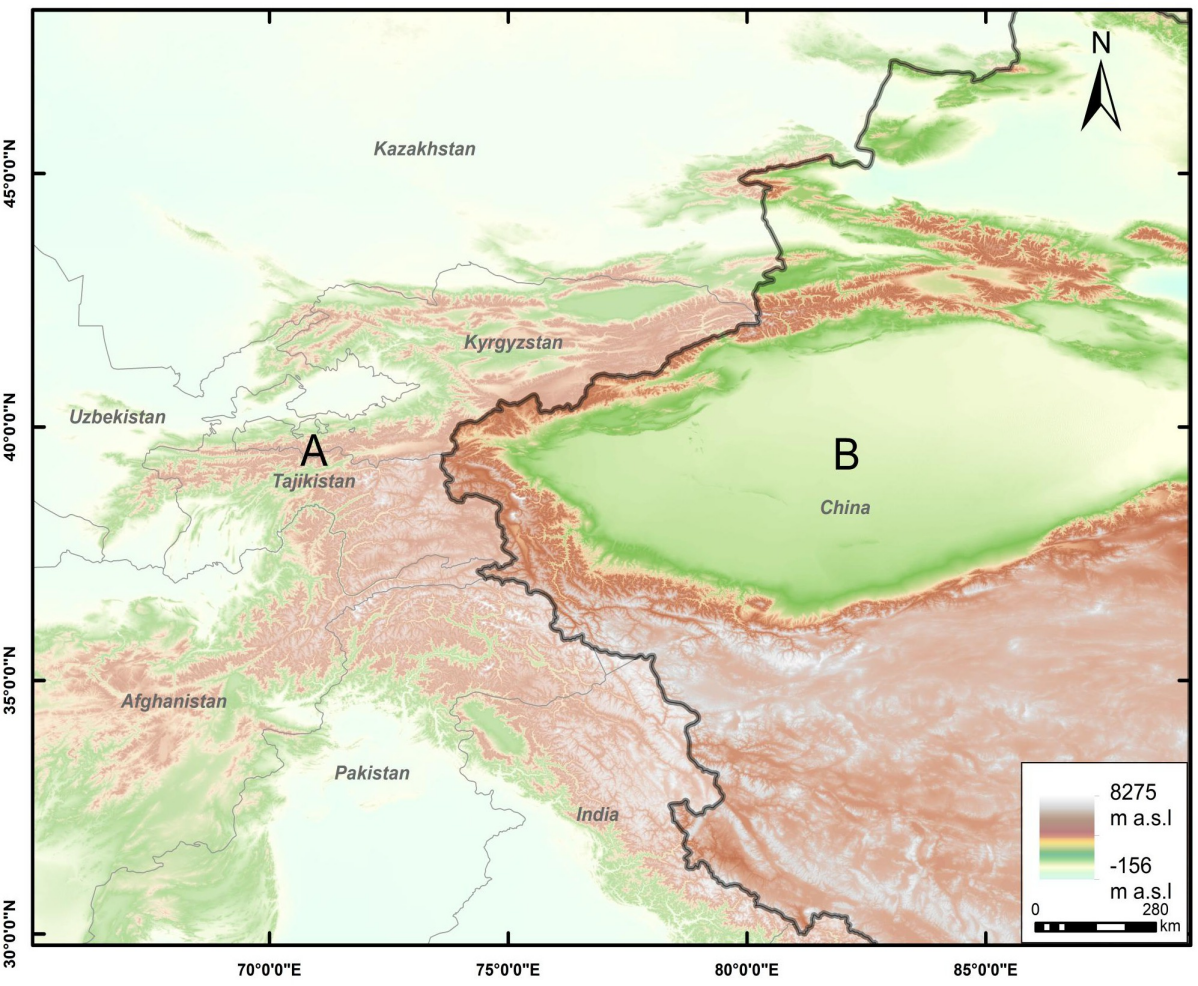
The study area. Elevation within the study area is depicted by the Digital Elevation Model (DEM). Boundaries obtained from Natural Earth (http://www.naturalearthdata.com/). DEM was obtained from USGS Earth Explorer (https://earthexplorer.usgs.gov). This schematic line illustrates the relative positions of each country and should not be reused or misinterpreted for any political purposes.

The study area (29.75–48.23 N, 64.98–89.25 E) covering approximately 4.30×10^6^ km^2^, includes seven countries, Afghanistan, China, India, Kyrgyzstan, Pakistan, Tajikistan, and Kazakhstan (Fig 1).

The western region of China mainly includes parts of the Tarim Basin and the Tibetan Plateau, characterized by a continental climate with significant variations in day and night temperatures as well as seasonal temperatures. In this region, temperature varies greatly with altitude, featuring dry and arid conditions, low humidity, and high evaporation rates. Precipitation levels are low, with most areas receiving less than 500 mm of annual precipitation. Precipitation is unevenly distributed, with more rainfall in mountainous regions and less in desert areas. Western China has a variety of soil types, including desert, loess, and mountain soils. Soils in the region are generally nutrient-poor and low in organic matter, making them prone to erosion and desertification. The study area also includes the Central Asian countries bordering China (Kazakhstan, Kyrgyzstan, Tajikistan, and Pakistan), which share a continental climate with cold winters and hot summers. Annual precipitation is low and is mainly distributed in spring and summer. Central Asia has a wide range of vegetation types, including steppes, shrublands, forests, and desert sand, and soil types vary from fertile river valleys to arid desert soils. The region also has large areas of permafrost. At the border of China, Tajikistan, and Afghanistan lies the Pamir Plateau, with an average altitude exceeding 4500 m a.s.l. Due to the diverse terrain, it is divided into the East Pamir Plateau and the West Pamir Plateau, features a continental alpine climate. The East Pamir Plateau experiences long winter, with an average temperature of -17.8°C in January and an absolute minimum temperature of -50°C at around 3600 m a.s.l, characterized by permafrost and salt soil.

### Research data collection and preprocessing

We obtained the occurrence points of FMD serotype O (n = 386, S1 Table) from published literature and reports from the World Organisation for Animal Health (WOAH). Four groups of environmental predictor variables were considered: climate, terrain, vegetation, and human impact (Table 1). The data layer was resampled to 30 arc-seconds when necessary, and the data were processed and calculated in ArcGIS 10.6 using the UTM-WGS-1984 projection coordinate system.

**Table 1:** The environmental predictor variables used to predict FMD serotype O. The second and third columns are the data source and data range and type, respectively.

Variable	Source	Variable value range or categories (type)
**Climate** ^a^		
Monthly P	Worldclim	0 to 1047 mm/month
Monthly meanT	Worldclim	-35.2 to 37.9 °C
Monthly minT	Worldclim	-41 to 30.8 °C
Monthly maxT	Worldclim	-29.3 to 44.9 °C
Bioclimatic(bio1-19)	Worldclim	Annual trends, seasonality, extreme or limiting environmental variables
**Terrain**		
Elevation	ASTER-GDEM^b^	-153 to 8275 m a.s.l
Slope angle	ASTER-GDEM	0 to 55.56°
Distance to river	ASTER-GDEM	0 to 2.77 km
**Human impact**		
Population	WorldPop^c^	0 to 68212.4 persons/km^2^
Sheep population density	FAO^d^	0 to 77994 individual/km^2^
Goat population density	FAO	0 to 74040 individual/km^2^
Cattle population density	FAO	0 to 33800 individual/km^2^
Buffalo population density	FAO	0 to 46903 individual/km^2^
**Vegetation**		
Land cover	ESA^e^	Categorical
Soil	HWSD	Categorical

^a^ T = temperature; P = precipitation. Source: https://worldclim.org/

^b^ Source: https://gdem.ersdac.jspacesystems.or.jp/

^c^ Source: https://www.worldpop.org/

^d^ Source: https://www.fao.org/livestock-systems

^e^ Source: https://maps.elie.ucl.ac.be/CCI/viewer/

^f^ Source: https://www.fao.org/soils-portal/data-hub/en/

### FMD spatial distribution model

The study area was divided into regions A and B according to the Chinese borderline. We treated regions A and B separately within the study area, due to their significant geographic elevation differences [23]. Based on the highland climate [24], models were constructed separately for high-elevation and low-elevation areas at each location below 1500 m a.s.l. and above 1500 m a.s.l. to improve model robustness [25].

Spatial autocorrelation between records affects model quality [26, 27]. To address this issue, we employed spatial rarefaction (Filtering) to decrease the number of records in the oversampling area [27, 28]. In the initial filtering, we applied the default distance setting (natural break), which ensures that the maximum distance between two adjacent points is less than 50 km and the minimum distance is greater than 5 km. We set the gradient’s minimum distance between each pair of existing point records, starting from 0 km and increasing in 5 km increments to filter out multiple groups with varying minimum distances. The group of distribution point data with different gradients as the minimum distance. The data were filtered using the SDM Toolbox v1.3 in ArcGIS.

Multicollinearity, particularly among highly correlated variables, can significantly impact the model [29, 30]. Climate as well as non-climate variables were treated to reduce their multicollinearity. First, we utilized principal component analysis (PCA) for the selection of the main predictors [31, 32]. Subsequently, we eliminated variables with low contributions and high standard deviations, guided by the MaxEnt model’s results [32–34]. We then performed Variance Inflation Factor (VIF) analysis on the filtered variables, considering those with a VIF below 10 as having low multicollinearity [29, 35].

We randomly divide the collected presence records into a 70% training part and a 30% test part to construct and test the model, using 10 bootstrap replicates. The default settings were retained for the remaining parameters. The FMD serotype O risk prediction maps obtained by fuzzy superimposed models A1, A2, B1 and B2. The model’s output was classified using the Jenks natural breaks optimization method [27, 36]. For enhanced visualization, smoothing techniques were applied to produce the final FMD serotype O risk prediction maps [26, 33].

### The least-cost path

The least-cost path (LCP) refers to the shortest route between the ‘origin’ and ‘destination’ and is the most efficient path for moving individuals [37]. It has been widely accepted as a valuable method for predicting disease transmission [25, 27, 35]. To create cost surfaces for ungulate movement, we considered elevation and land cover as cost factors, aligning with their movement preferences. The primary data for landcover layers consist of classified layers, while elevation data are categorized using the Jenks natural breaks method [25, 27, 35, 38]. The resistance values assigned to these surfaces range from 1 to 9, where higher values indicate greater resistance to animal movement across the habitat.

The raster representing grassland, forest, scrub, and mosaic plant types had the lowest cost value (= 1) to reflect their favorability for ungulate movement [25, 39]. Conversely, terrain features unfavorable for passage, such as water bodies, lakes, permanent snow, ice, and bare areas, were assigned the highest cost value (= 9) [25, 27]. The locations of FMD serotype O outbreaks are observed to be clustered. LCP between FMD serotype O clusters is analyzed using the cost surface of the build. Remove the move away from the boundary, and take the inner path to reach the final crossover path.

## Results

**Fig 2:**
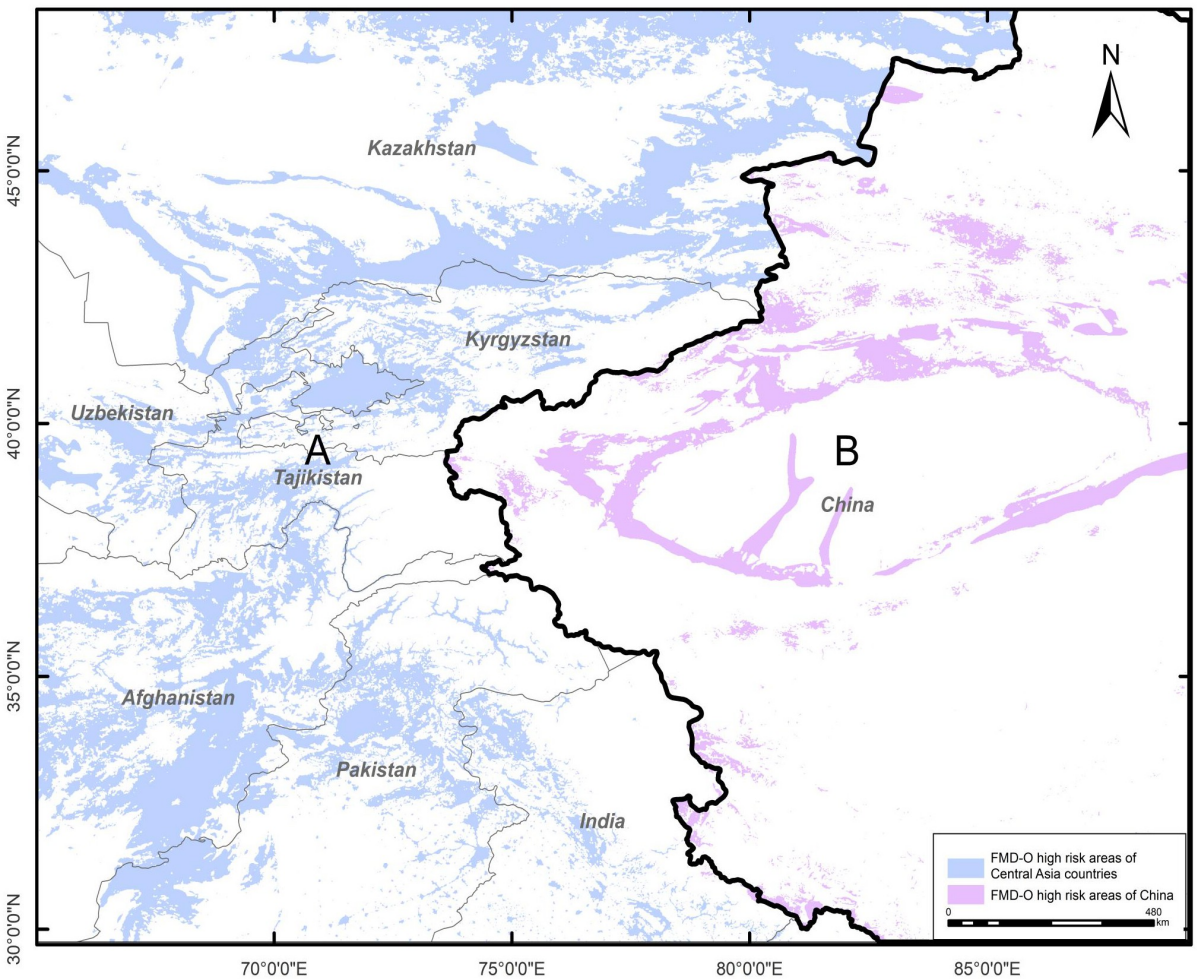
FMD serotype O high-risk areas predicted by the MaxEnt model. This resulting map was created in ArcGIS 10.6 from raster results generated by MaxEnt. Boundaries obtained from Natural Earth (http://www.naturalearthdata.com/). This is a schematic line illustrating the relative position of each country and should not be reused or misinterpreted for any political reason.

Areas at high risk of FMD serotype O (Fig 2) are mainly located in eastern and western Kazakhstan, northern Pakistan, eastern Afghanistan, western Tajikistan, northern Xinjiang, China, and Xinjiang bordering India.

### Results of FMD spatial distribution models

#### Model A1 (≥1500 m)

**Fig 3:**
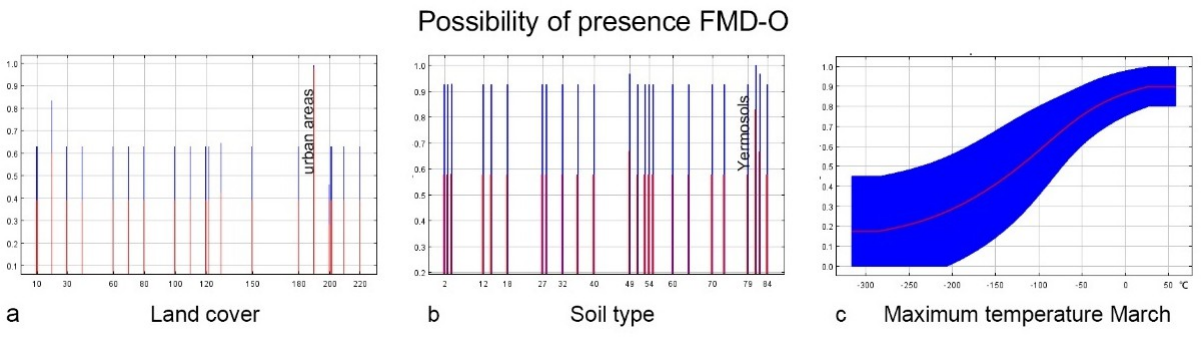
The response curves of Model A2. The curves show the mean response (red) and the mean standard deviation (blue).

Model A1 was constructed using 4 geolocation data points of at least 10 km after screening. After screening by the stepwise elimination method, three environmental factors with a contribution of more than 10% were obtained, namely land cover, distance to the river, and Mean Diurnal Range (Mean of monthly (max temp—min temp)). The VIF values of the three variables ranged from 1.018 to 1.179, indicating low multicollinearity. Moreover, the value of AUC was 0.913, and the Standard Deviation (SD) was 0.045, indicating high robustness of the model. The response curves and contribution rates of the three predictor variables are shown in Fig 3 and Table 2 (right), respectively.

**Table 2:** The contribution of variables in Models A1 and A2.

ModelA1(≥1500)			ModelA2(<1500)		
Variable	Contribution%	Permutation importance	Variable	Contribution%	Permutation importance
Mean Diurnal Range	44.6	55.7	Land cover	52	22.9
Land cover	44.2	13	Soil	31.1	29.8
Distance to river	11.2	31.3	Min T Mar	16.9	47.3

#### Model A2 (<1500 m)

**Fig 4:**
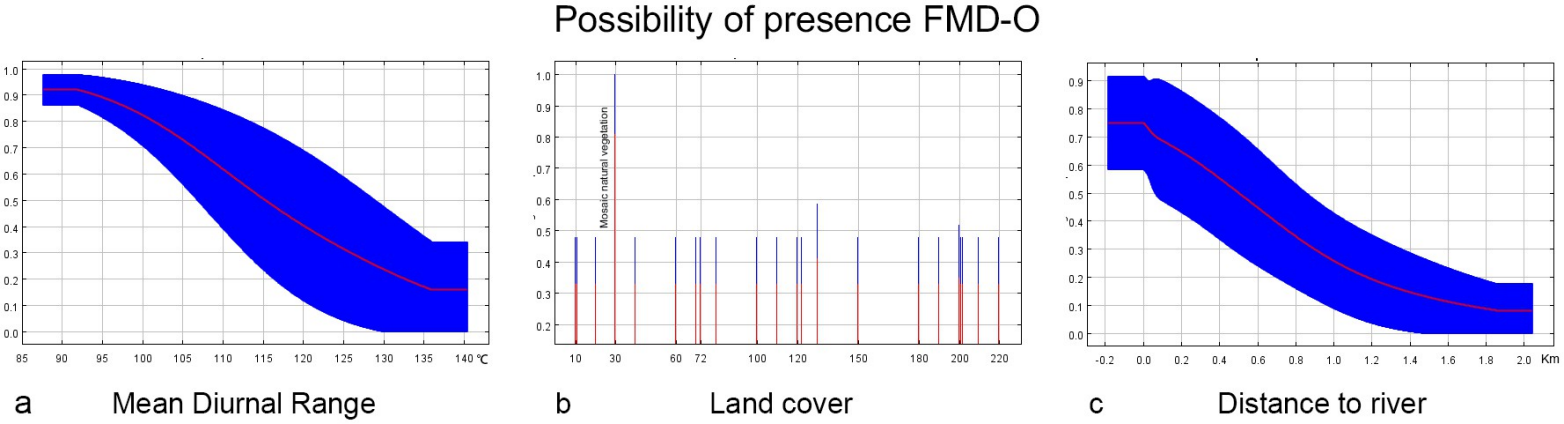
The response curves of Model A1. The curves show the mean response (red) and the mean standard deviation (blue).

To reduce the potential spatial autocorrelation, 41 geolocation data points were obtained after filtering the raw data, which were at least ten kilometers apart from each other. After screening through principal component analysis and the stepwise elimination method, three environmental factors with a contribution of more than 10% were obtained, namely Min T Mar, soil type, and land cover. The three predictor variables VIF values are in the range of 1.001–1.335, all less than 10. The AUC value of the model was 0.972, and the SD value was 0.012. The response curves and contribution rates of the three predictor variables are shown in Fig 4 and Table 2 (left), respectively.

#### Model B1 (≥1500 m)

**Fig 5:**
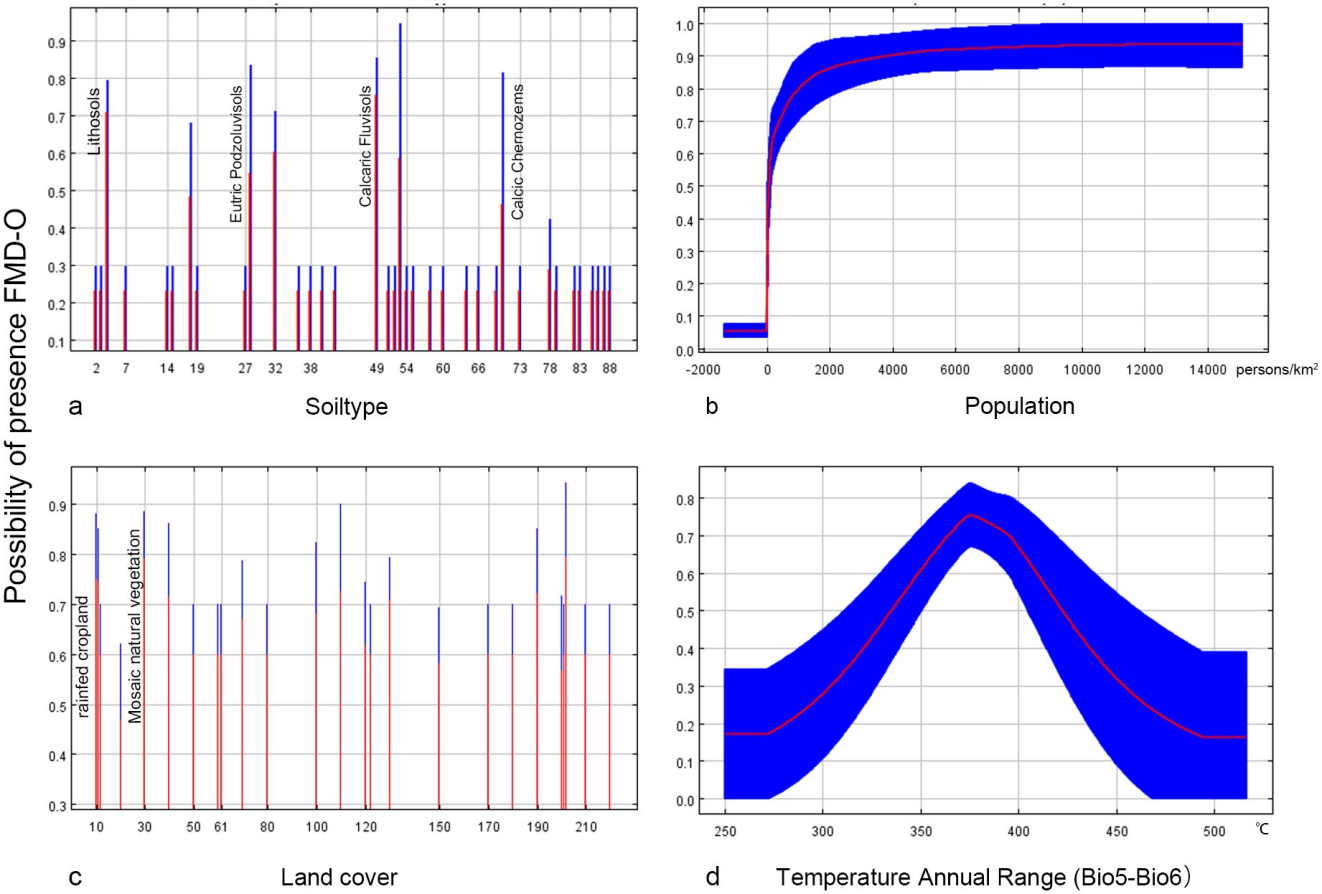
The response curves of Model B1. The curves show the mean response (red) and the mean standard deviation (blue).

Model B1 was constructed using 232 geolocation data points, each at least 10 km after screening. After screening using the stepwise elimination method, four environmental factors with a contribution of more than 10% were identified: soil types, human population, land cover, and Temperature Annual Range. The VIF values of the three variables ranged from 1.018 to 1.307, indicating low multicollinearity. Moreover, the value of AUC was 0.866, and the value of SD was 0.012, indicating high robustness of the model. The response curves and contribution rates of the three predictor variables are shown in Fig 5 and Table 3 (left), respectively.

**Table 3:** The contribution of variables in Models B1 and B2.

ModelB1(≥1500)			ModelB2(<1500)		
Variable	Contribution %	Permutation importance	Variable	Contribution %	Permutation importance
Soil type	35.5	30.5	Land cover	28	14.3
Human Population	33.8	31.2	Human Population	26.4	22.5
Land cover	18.7	13.6	Max T June	24.4	49
Temperature Annual Range	12.1	24.7	Soil type	21.2	14.2

#### Model B2 (<1500 m)

**Fig 6:**
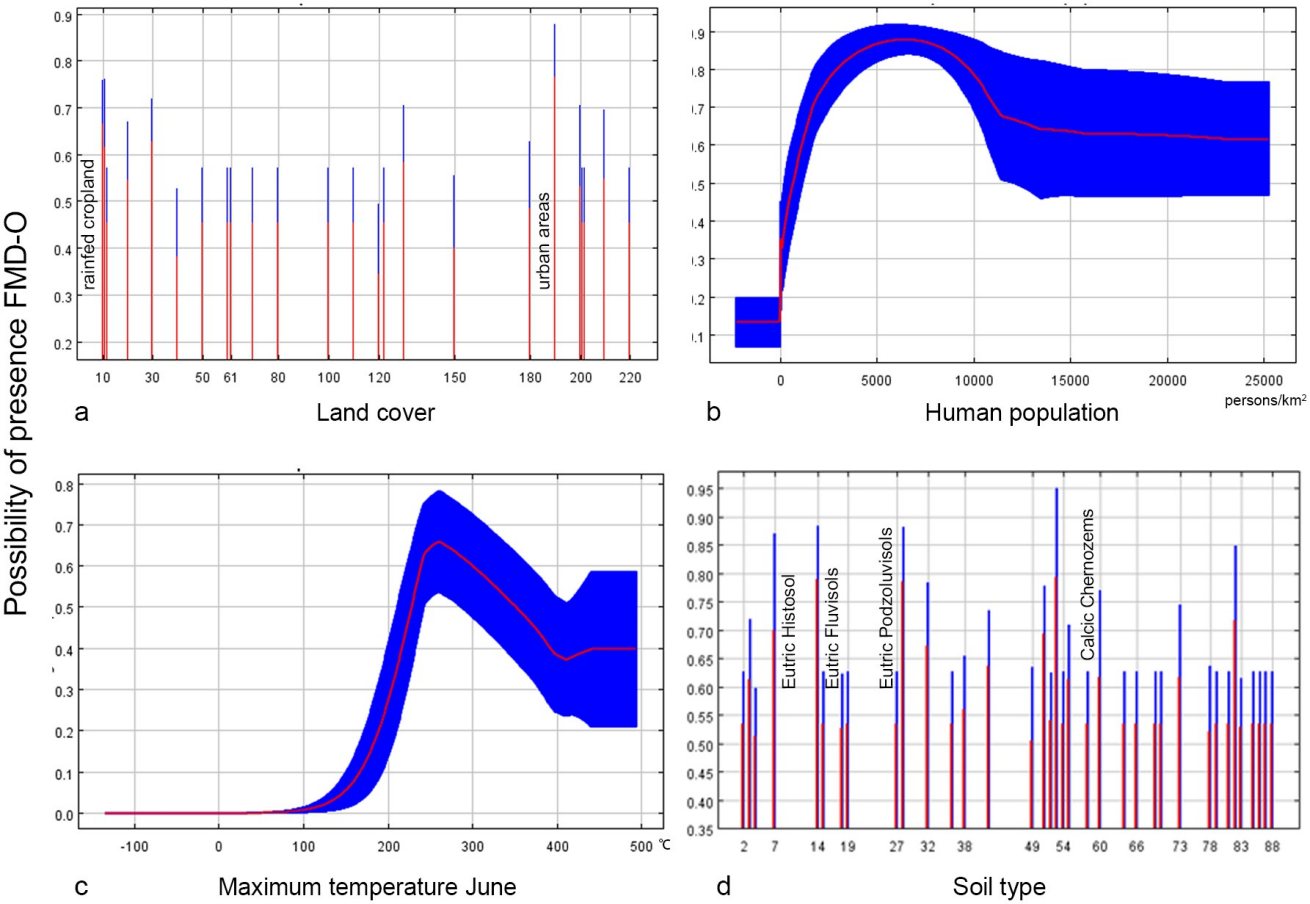
The response curves of Model B2. The curves show the mean response (red) and the mean standard deviation (blue).

Model B2 was constructed using 42 geolocation data points of at least 10 km after screening. After screening by the stepwise elimination method, four environmental factors with a contribution of more than 10% were identified: land cover, human population, Max T June, and soil type. The VIF values of the three variables ranged from 1.030 to 1.264, indicating low multicollinearity. Moreover, the value of AUC was 0.916, and the value of SD was 0.011, indicating high robustness of the model. The response curves and contribution rates of the three predictor variables are shown in Fig 6 and Table 3 (right), respectively.

### Potential risk pathways

**Fig 7:**
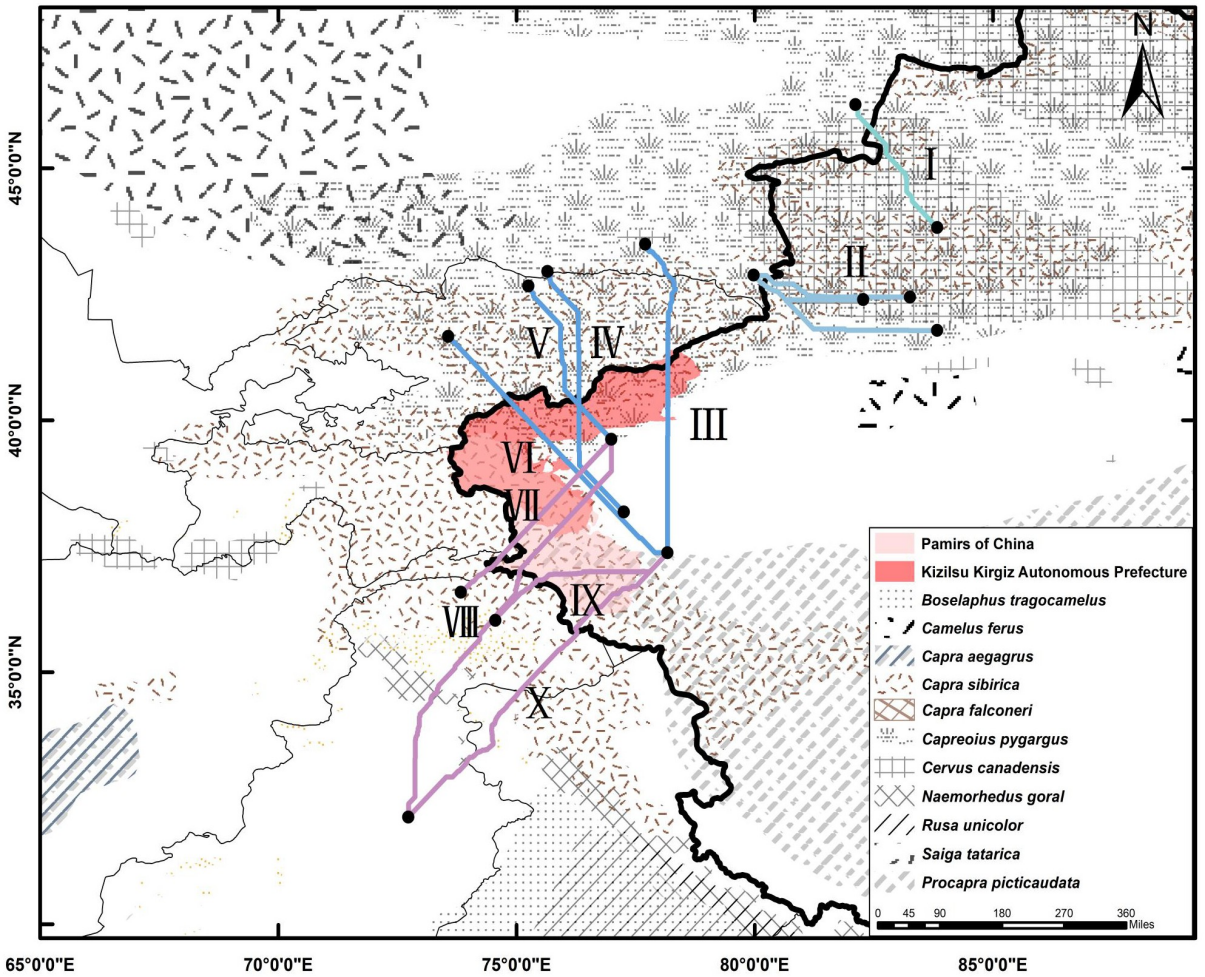
Transboundary LCPs and the distribution of ungulates. Boundaries obtained from Natural Earth (http://www.naturalearthdata.com/). This is a schematic line illustrating the relative position of each country and should not be reused or misinterpreted for any political reason. The territory range of wild ungulates was obtained from International Union for the Conservation of Nature (IUCN) website (https://www.iucnredlist.org/). The data used for this figure is under CC BY license, and permission for its use has been obtained from the IUCN.

Using LCP analysis, we identified 10 potential risk pathways for the transmission of FMD serotype O within the four study areas (Fig 7).

I. Kazakhstan—Ili Kazak Zizhizhou (Xinjiang, China);
III. Kazakhstan—Aksu Diqu (Xinjiang, China);
III. Kazakhstan—Hotan area (Xinjiang, China);
IV. Kazakhstan—Kashgar (Xinjiang, China);
V. Kyrgyzstan—Kashgar (Xinjiang, China);
VI Kyrgyzstan—Hotan area (Xinjiang, China);
VII. Pakistan—Kashgar (Xinjiang, China);
VIII. Pakistan—Kashgar (Xinjiang, China);
IX. Pakistan—Hotan area (Xinjiang, China);
X. Pakistan—Hotan area (Xinjiang, China).

## Discussion

Temperature plays an crucial role in the distribution of FMDV and the temperature factor was retained in all four models (A1 = 44.6%, A2 = 16.9%, B1 = 12.1%, B2 = 24.4%). In the high-altitude model, FDMV is sensitive to drastic temperature changes. The annual temperature range (35–40°C) and the mean diurnal range (8.5–10°C) are more favorable according to the response curve. The greater the temperature difference, the lower the risk. The response curves suggest that at low altitudes, minimum temperatures of 2–5°C in March and maximum temperatures of 23–30°C in June may favor FMDV survival. The virus survival was more favorable at low temperatures with low relative humidity and at high temperatures with high relative humidity, possibly due to microclimatic conditions [40]. These findings underline the importance of considering FMD transmission seasonality in prevention and control strategies.

Land cover variables played a significant role in all four models, with high contribution percentages (A1 = 44.2%, A2 = 22.9%, B1 = 52%, B2 = 28%). Habitats with deciduous rainfed cropland, urban areas, or mosaics of natural vegetation (tree, shrub, herbaceous cover) (>50%)/cropland (<50%) had the highest probability of hosting FMD serotype O, as indicated by the response curve. At high altitudes within the study area, grasslands and mixed vegetation types are prevalent, providing key habitats for sheep and goats, which are mainly raised in these pastures. In these high-altitude areas, mixed vegetation not only supports livestock but also provides food and shelter for wild ungulates. Furthermore, both urban areas and rainfed croplands, associated with higher human activities, emerged as relevant in our analysis.

It’s documented that under optimal conditions of temperature, relative humidity, and pH, FMDV’s median survival period in the soil is 23.5 days, ranging from 2 to over 30 days [40]. Soil type played a significant role in three models (A2 = 31.1%, B1 = 35.5%, B2 = 21.2%). Yermosols has a high correlation in low-altitude areas in China. This can be explained by the fact that the reported low—altitude cases in China are distributed around the Tarim Basin, where the dominant land type is desert [41]. Four soil types, Lithosols, Eutric Podzoluvisols, Calcaric Fluvisols, and Calcic Chernozems, show a high correlation in high-altitude areas of Central Asia. Soils rich in calcium carbonate and organic matter appear to influence FMD serotype O survival at these altitudes. At low altitudes in Central Asia, common soils like Eutric Histosols, Eutric Podzoluvisols, Eutric Fluvisols, and Calcic Chernozems, are eutrophic and fertile soil types with high amounts of organic matter. The complexity of the soil matrix, its texture and structure, as well as the electrical charge of the soil, which gives it various physical and chemical properties, can affect the movement of microbes within the system [40]. Further study may be required to investigate the specific effects of different soil substrates on.

We incorporated various terrain variables to aid in predicting the risk of FMD serotype O. Elevation and slope are critical factors influencing ungulate range and, consequently, FMD serotype O spread [42, 43]. In our high-altitude model for western China, FMD serotype O spread risk shows a negative correlation with the distance to river sources. This is notable considering FMD’s median survival of 28.5 days in water, with a range of 11 to 30 days [40]. The distance to lakes also influenced the distribution of most Tibetan Plateau ungulates [43]. Water sources like lakes and rivers are crucial for supporting habitats and the life processes of animals, potentially leading to ungulate aggregation. This is one of the possible causes of the spread of FMD [44]. In our model, slope was not a significant factor as ungulates generally possess strong climbing abilities, rendering slope less restrictive in their distribution [45].

The spread of FMD was highly correlated with human activities, including transportation and trade [46]. Population density was positively correlated with FMD serotype O in our model in high and low altitude patterns in Central Asian countries, except for western China, characterized by high altitude, complex terrain, and low population density [47]. Traditional Muslim festivals in Central Asian countries, like Eid al-Fitr, often involve extensive animal trade and movement, increasing the risk of FMD serotype O transmission through animal products [48]. Although livestock population made a low contribution to our model, this doesn’t negate its impact on FMD serotype O transmission. This is likely due to a high correlation between livestock density and population density variables [27].

The LCP analysis returned ten cross-border paths between Kazakhstan, Kyrgyzstan, Pakistan and China.

Path I starts at Lake Alakol in East Kazakhstan Oblast, traversing the China-Kazakhstan border into the Ili Autonomous Prefecture in Xinjiang, China. This path mainly follows river valleys and lakes, facilitating easier crossings for ungulates [49]. Path II begins in Taldykorgan, Kazakhstan, and reaches the Aksu region from the base of Jengish Chokusu. This route is identified as having a higher risk for FMD serotype O transmission. Originating south of Kapchagai Reservoir in Kazakhstan, Path III runs along the Trans-Ili Mountains, encircles Issyk-Kul Lake, passes through the Biedieli Pass, and enters Xinjiang.

Path IV, V and VI all enter China from Kizilsu Kirgiz Autonomous Prefecture, Xinjiang Province. Path IV starts in the valleys of the Trans-Ili and Kyrgyz Mountains, entering Xinjiang from the southern Tianshan Mountains. It traces a significant wildlife and livestock-rich corridor. Similar to Path IV, Path V also commences in Kyrgyzstan, following a parallel trajectory into Xinjiang. Originating from Kiziloy in Kyrgyzstan, Path VI extends along the Naren River into the Hotan region of Xinjiang. This path, rich in vegetation, provides an ideal habitat for diverse wildlife [49], and is also a hub for animal husbandry, a predominant industry in the region [50]. According to government statistics, the Kizilsu Kirgiz Autonomous Prefecture had 2,151,800 livestock heads by the end of 2022, mainly cattle and sheep [50]. The traversal of these paths through livestock-dense areas highlights the critical role of animal husbandry in facilitating FMD transmission.

Path VII, VIII, IX, and X originate in Pakistan and cross the Pamir Plateau, leading to the Kashgar and Hotan regions in China. The Pamir Plateau, ranging from 3500 to 5500 m a.s.l above sea level, is characterized by its rocky, mountainous terrain [51]. This plateau encompasses the Taxkorgan Nature Reserve, which is the junction of biodiversity hotspot in China [52]. A variety of wild ungulates such as the Siberian ibex (*Capra sibirica*) [53] and Marco Polo sheep (*Ovis ammon polii*) [54]. Despite the high elevations, particularly in Path X, the region’s topography facilitates FMD transmission among animals. This underscores how animal species and their densities along these LCP routes might influence FMD virus transmission pathways. Moreover, the Pamir plateau is also an important potential transmission area in the prediction of transboundary transmission of peste des petits ruminants [25]. Effective surveillance of FMD outbreaks in this area is crucial for interrupting cross-border disease spread.

Susceptible wildlife populations and uncontrolled animal movement are key risk factors for FMD, with seasonally associated cross-border animal grazing particularly challenging FMD control efforts in Africa [55–57]. Antibodies to FMDV or clinical disease have been observed in numerous species such as *Saiga tatarica* [58], *Procapra gutturosa* [59], roe deer (*Capreolus capreolus*), and red deer (*Cervus elaphus*) [60]. The number of local veterinarians [61], livestock markets [62], and special populations (such as artificial inseminators and farm veterinarians) [63] may also have an impact on FMD transmission. Obtaining detailed dynamic data layers is challenging due to animal migration and population movements. However, key factors influencing animal movement and grazing, such as precipitation, temperature, vegetation, population density, and livestock density, are accounted for in our MaxEnt model. Additionally, we incorporated population data to analyze the relationship between wildlife and FMD. The wide distribution of FMD wildlife hosts, especially the Siberian ibex (*Capra sibirica*) in almost every pathway, may provide enough wild hosts for the spread of FMD.

Utilizing multiple environmental variables, a MaxEnt model was developed to elucidate the risk factors and high-risk areas for FMD serotype O in western China and the neighboring regions of Central Asian countries along the Chinese border. Only 17 cases of FMD serotype O were reported from the western border of China, providing valid data for modeling purposes. It has been shown that MaxEnt modeling is more accurate in the prediction of small sample sizes [64]. In our LCP model, we focused on primary factors influencing ungulate movement and survival, such as altitude and vegetation. Secondary factors like natural enemies, hunting, and detailed landscape habitat use were not included due to their quantitative measurement [27]. This is because they are difficult to measure and use quantitatively. Notably, LCP proves to be an effective and versatile quantitative method, especially useful in large-scale areas or regions where field monitoring is challenging [27, 65].

The LCP analysis in our study is pivotal for identifying probable routes of FMD serotype O transmission across diverse landscapes, factoring in the geographical and environmental influences on ungulate movement. This insight not only facilitates targeted surveillance and control in regions where FMD poses significant risks to health and the economy but also aids in direct observation in challenging areas. Additionally, given FMD’s transboundary nature, LCP is instrumental in pinpointing key areas for international cooperation and coordinated response, crucial for disease control across national borders.

In conclusion, our study reveals that the distribution of FMD serotype O is influenced by a diverse array of climatic, land cover, soil, terrain, and anthropogenic factors, underscoring the need for a multidisciplinary approach to effectively control FMD. As part of a global initiative to eliminate FMD, we modeled and assessed the risk of FMD serotype O in western China and its bordering regions [66]. Our findings indicate seasonal variations in FMD serotype O survival at low altitudes (notably in March and June), with a heightened sensitivity to temperature differences at higher altitudes. The risk of FMD serotype O was particularly high in Central Asian countries, closely correlating with population variables. Ten cross-border LCP paths representing Pakistan, Kazakhstan, Kyrgyzstan, and China were predicted. Notably, the Pamir Plateau and Kizilsu Kirgiz Autonomous Prefecture in Xinjiang, China, emerged as potential transboundary hotspots for FMD serotype O. This study will provide guidance in monitoring the FMD serotype O epidemic and preventing its cross-border spread in the future.

## Supporting information

S1 Table The geolocations of FMD serotype O. (CSV)
